# Neural complexity through a nonextensive statistical–mechanical approach of human electroencephalograms

**DOI:** 10.1038/s41598-023-37219-5

**Published:** 2023-06-26

**Authors:** Dimitri Marques Abramov, Constantino Tsallis, Henrique Santos Lima

**Affiliations:** 1 Laboratório de Neurobiologia e Neurofisiologia Clínica, Instituto Nacional da Saude da Criança, da Mulher e do Adolescente Fernandes Figueira, Fundacao Oswaldo Cruz, Avenida Rui Barbosa 716, Flamengo, Rio de Janeiro, 22250-020 Brazil; 2 Centro Brasileiro de Pesquisas Fisicas and National Institute of Science and Technology for Complex Systems, Rua Xavier Sigaud 150, Rio de Janeiro, 22290-180 Brazil; 3 Santa Fe Institute, 1399 Hyde Park Road, Santa Fe, NM 87501 USA; 4 Complexity Science Hub Vienna, Josefstädter Strasse 39, 1080 Vienna, Austria

**Keywords:** Applied physics, Biological physics, Statistical physics, thermodynamics and nonlinear dynamics

## Abstract

The brain is a complex system whose understanding enables potentially deeper approaches to mental phenomena. Dynamics of wide classes of complex systems have been satisfactorily described within *q*-statistics, a current generalization of Boltzmann-Gibbs (BG) statistics. Here, we study human electroencephalograms of typical human adults (EEG), very specifically their inter-occurrence times across an arbitrarily chosen threshold of the signal (observed, for instance, at the midparietal location in scalp). The distributions of these inter-occurrence times differ from those usually emerging within BG statistical mechanics. They are instead well approached within the *q*-statistical theory, based on non-additive entropies characterized by the index *q*. The present method points towards a suitable tool for quantitatively accessing brain complexity, thus potentially opening useful studies of the properties of both typical and altered brain physiology.

## Introduction

The brain is widely recognized as a complex system since it is composed by billions of cells (neurons) which express individual behaviors and, at same time, they build a fully interconnected network with emergent, self-organized collective behaviors^1^. Thus, traditional reductionist scientific methodology from mechanistic rationality appears to fail for deeply understanding the brain and its associated mind inside a multidimensional environment^2^. On one hand, a humanity’s great unresolved problem is to establish a suitable mental medicine, from epistemology^3^ to the biomedical perspective. The problem begins in differentiating normality from typicality, illness from neurodiversity. And, upon this basis, to establish a taxonomy about mental typology for a more realistic nosography. On the other hand, several studies have explored brain complexity through entropic measures within the electroencephalogram (EEG), and found relationships between brain complexity and different mind conditions^4^. However, this issue yet is incipient. One way of accessing brain complexity is through the electroencephalogram (EEG) signal^5^, which is the electrical result of millions of neurons under each of the leads (electrodes) over time. The EEG is the simplest, least invasive and universally used form of functional recording of the human brain dynamics.

The pioneering works of Boltzmann^6^ and Gibbs^7^ (BG) established a magnificent theory which is structurally associated with the BG entropic functional

1 $$\begin{aligned} S_{BG}=-k\sum _{i=1}^W p_i \ln p_i \;\;(\sum _{i=1}^W p_i=1), \end{aligned}$$

and consistent expressions for continuous or quantum variables; *k* is a conventional positive constant (in physics, *k* is chosen to be the Boltzmann constant $k_B$; in information theory and computational sciences, $k=1$ is frequently adopted).

In the simple case of equal probabilities, this functional becomes $S_{BG}=k \ln W$. Equation (1) is generically *additive*^8^. Indeed, if *A* and *B* are two probabilistically independent systems (i.e., $p_{ij}^{A+B}= p_i^A p_j^B$), we straightforwardly verify that $S_{BG}(A+B)=S_{BG}(A)+S_{BG}(B)$. The celebrated entropic functional (1) is consistent with thermodynamics for all systems whose *N* elements are either independent or weakly interacting in the sense that only basically local (in space/time) correlations are involved. For example, if we have equal probabilities and the system is such that the number of accessible microscopic configurations is given by $W(N) \propto \mu ^N\; (\mu >1; \,N\rightarrow \infty )$, then $S_{BG}(N)$ is *extensive* (i.e., proportional to the number of elements) as required by thermodynamics. Indeed $S_{BG}(N)=k\ln W(N) \sim k(\ln \mu )N$.

However, complex systems are typically composed of many elements which essentially are non-locally correlated, building an intricate network of interdependencies from where collective states can emerge^9^. BG statistical mechanics appears to be generically inadequate for such systems because this theory assumes (quasi) independent components with short-range (stochastic or deterministic) interactions.

Indeed, if the correlations are nonlocal in space/time, $S_{BG}$ may become thermodynamically inadmissible. Such is the case of equal probabilities with say $W(N) \propto N^\nu \;(\nu >0; \,N\rightarrow \infty )$: it immediately follows $S_{BG}(N) \propto \ln N$, which violates thermodynamical extensivity^9^. To satisfactorily approach cases such as this one, it was proposed in 1988^10^ to build a more general statistical mechanics based on the *nonadditive* entropic functional

2 $$\begin{aligned} S_q\equiv k\frac{1-\sum _{i=1}^W p_i^q}{q-1}=k\sum _{i=1}^W p_i \ln _q \frac{1}{p_i} = -k\sum _{i=1}^W p_i^q \ln _q p_i = -k\sum _{i=1}^W p_i \ln _{2-q} p_i \;\;(q \in {\mathbb {R}}; S_1=S_{BG}), \end{aligned}$$

with the *q*-logarithmic function $\ln _q z \equiv \frac{z^{1-q}-1}{1-q} \; (\ln _1 z=\ln z)$, its inverse being the *q*-exponential $e_q^z \equiv [1+(1-q)z]_{+}^{1/(1-q)}$; $(e_1^z=e^z$; $[z]_{+}=z$ if $z>0$ and vanishes otherwise); for $q<0$, it is necessary to exclude from the sum the terms with vanishing $p_i$. We easily verify that equal probabilities yield $S_q=k\ln _q W$. Also, we generically have the following functional nonadditivity

3 $$\begin{aligned} \frac{S_q(A+B)}{k}=\frac{S_q(A)}{k}+\frac{S_q(B)}{k}+(1-q)\frac{S_q(A)}{k}\frac{S_q(B)}{k} . \end{aligned}$$

Consequently, in the $(1-q)/k \rightarrow 0$ limit, we recover the $S_{BG}$ additivity. For the anomalous class of systems mentioned above, namely if $W(N) \propto N^\nu$, we obtain, $\forall \nu$, the *extensive* entropy $S_{1-1/\nu }(N)=k\ln _{1-1/\nu }W(N) \propto N$, as required by the Legendre structure of thermodynamics^11,12^. Finally, the optimization of $S_q$ under simple constraints yields *q*-exponential distributions for the (quasi)stationary states, instead of the usual BG exponentials.

**Figure 1:**
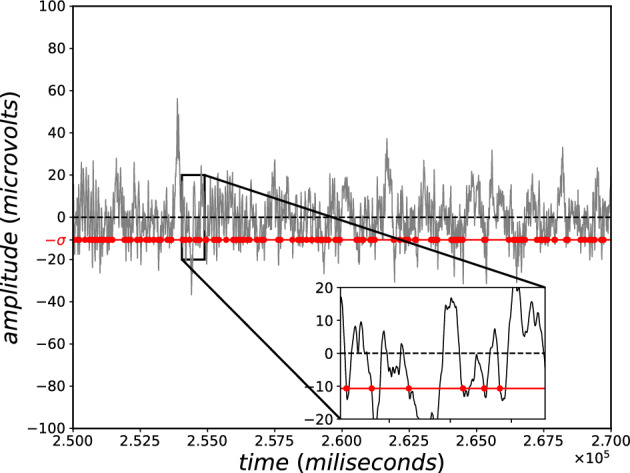
Segment of ongoing EEG from one subject (B006), recorded on the mid-parietal ($P_z$) location of the head. Red dots: time values when ddp (signal amplitude) crosses downwards the bottom threshold (1.0 standard deviation; red line). EEG sampling rate was 1000 Hz.

**Figure 2:**
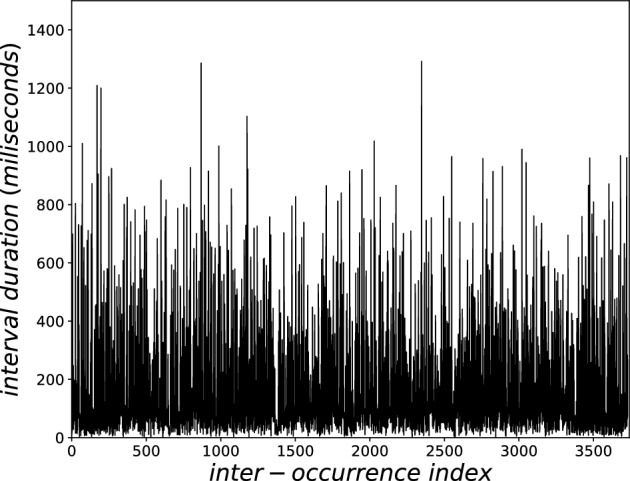
Sequence of inter-event time intervals from EEG signal, as detected in Fig. 1.

Since EEG is a massive electrical phenomenon, its amplitude is correlated with the cell synchronization. The regularity of time intervals between amplitude peaks that overcomes a typical threshold (in this case, one standard deviation), would reflect the system’s complexity. If synchronization would be a stochastic and uncorrelated phenomenon, the distribution of inter-peak distances could possibly be estimated within the BG frame. But EEG is a highly non-equilibrium phenomenon, and it requires more general approaches. Independently of the nature of regularities, this phenomenon exhibits the complex nature of the system. It cannot be excluded that, in the realm of *q*-statistics where *q* is a scalar measure of complexity, a possibly satisfactory description could be attained.

## Motivation, methodology and results

The above nonadditive entropies, as well as the nonextensive statistical mechanics grounded on them, have been already used to characterize various aspects of complexity. Various data obtained from EEG, magnetoencephalograms (MEG), electrocardiograms (ECG), and others, have been analyzed in connection to *q*-statistics^13–16^. However, the discussion frequently focuses on qualitative ingredients. Our aim here is to demonstrate that nonextensive statistical mechanics is applicable to the brain as a complex system, thus providing specific values for the relevant parameters. Thus, we are analyzing human EEG’s in a specific manner herein described which eventually provides a small number of real numbers (such as *q*) having the potential of satisfactorily characterizing different regions of the brain, different functional neuro-states, nosologically different classes of human phenomenologies.

**Figure 3:**
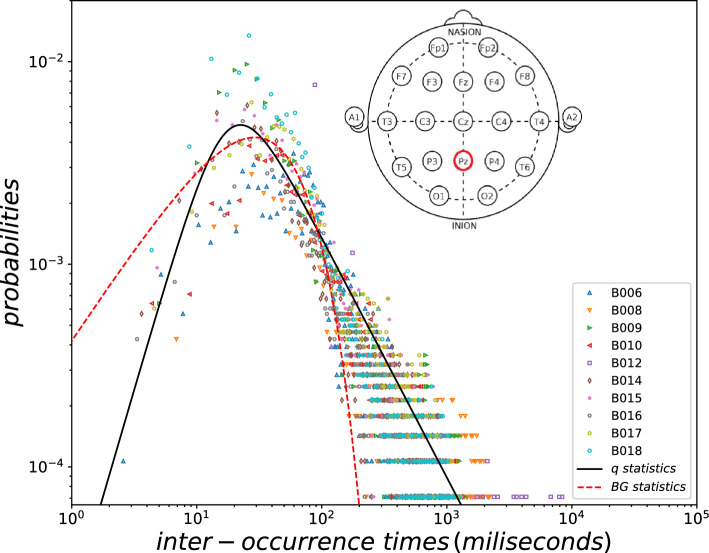
Probability distributions of EEG inter-occurrence times (500 equal logarithmic bins) and fittings with statistical models. Superimposed signal recorded on the $P_z$ location of ten subjects performing a work memory task. Amplitude threshold = 1.0 standard deviation. Fitting within Boltzmann-Gibbs statistical mechanics for non-complex systems (i.e., $q=1$, dashed red curve). Fitting within nonextensive statistical mechanics for complex systems (i.e., $q\ne 1$, black continuous curve). See Methodology for details.

We analized the EEG signal of ten typical adult humans from a match-to-sample task experiment with neutral affective interference for access working memory and attention, such in Yang and Zhen’s study^17^. This work was approved by our ethical board for human research, under CAAE 50137721.4.0000.5269. Each EEG signal has 5–10 min length recorded with open eyes at 1000Hz sampling rate, through 20 channels disposed at 10–20 montage with eyes open. The high, low and band-pass filters were respectively 0.5, 150 Hz and 60 Hz. We did not apply any other filter to minimize signal manipulation.

We accessed signal recorded at the midparietal ($P_z$) site (see Fig. 3), where classical cognitive event-related potentials, as P300^18^, manifest during attention tasks. A threshold was set at − 1.0 standard deviation from $P_z$ signal average (Fig. 1, from subject B006). Taking negative voltages we are minimizing the effect of blink artifacts, which are positive waves, amplier in frontal places.

Each event is the numerical position *i* of signal vector (1 s = 1000 positions) where the amplitude crossed the threshold downwards. The inter-event distances $i_n - i_{n-1}$ (where n = 1,...,N) were calculated (Fig. 2, from B006). The logarithm distribution of inter-event distances (with 500 distance classes) of all ten EEG signals at $P_z$ were superimposed, and the fitting was performed to the following *q*-statistical function (Fig. 3):

4 $$\begin{aligned} y_q=a_q\,x^{\,c_q}/[1+(q-1)\beta _q \,x^{\,\eta _q}]^{\frac{1}{q-1}}, \end{aligned}$$

where $(a_{q},\beta _{q},c_{q},\eta _{q},q)=(2.1 \times 10^{-5},2.0 \times 10^{-5},2.12,2.96,1.89)$ for the best fitting. And, for comparison, we also included the classical statistical BG function (where $q=1$), as follows:

5 $$\begin{aligned} y_{BG}=a_{BG}\,x^{\,c_{BG}}\,e^{-\beta _{BG} \,x^{\,\eta _{BG}}}. \end{aligned}$$

where $(a_{BG},\beta _{BG},c_{BG},\eta _{BG},q_{BG})=(4.3 \times 10^{-4},0.023,0.94,0.93,1)$ for the best fitting.

The fitting was performed using three different methods: dog leg trust region^19^, trust region reflective^20^ and crow search^21^ algorithms, all available in Scipy library.

The constant *a* is determined by imposing normalization, i.e., $\int _0^\infty dx\,y(x)=1$. Consequently,

6 $$\begin{aligned} a_q^{-1}=\int _0^{\infty } dx\,\frac{x^{c_q}}{[1+(q-1)\beta _q x^{\eta _q}]^{\frac{1}{q-1}}}=(\beta _q(q-1))^{-\frac{c_q+1}{\eta _q}}\frac{\Gamma (\frac{1+c_q}{\eta _q})\Gamma (\frac{1}{q-1}-\frac{1+c_q}{\eta _q})}{\eta _q\Gamma (\frac{1}{q-1})} \end{aligned}$$

for $q>1$ and $\frac{1}{q-1}-\frac{1+c_q}{\eta _q}>0$. In the $q\rightarrow 1$ limit, we obtain

7 $$\begin{aligned} a_{BG}^{-1}= \frac{\beta _{BG}^{-\frac{c_{BG}+1}{\eta _{BG}}} \Gamma \left( \frac{c_{BG}+1}{\eta _{BG}}\right) }{\eta _{BG}}. \end{aligned}$$

It is observed that EEGs at $P_z$ position from all subjects express very similar distributions of distances. The EEG regularity was modelled by the *q*-statistics function instead BG one (Fig. 3).

## Discussion

Consistently with the use of $S_q$ entropy in numerous articles as a measure of complexity in neural systems, we believe that we bring here the demonstration of the applicability of non-extensive statistical mechanics on the collective behavior of a neural system through the regularities of EEG. This preliminary study exhibits as a proof of concept that *q*-statistics easily can quantitatively reveal some aspects of brain complexity through the *q* parameter. Future research needs to be carried out to determine whether this measure will be sensitive enough to discriminate the complexity of different regions or different states of the brain, as well as aspects of inter-individual diversity (among them, brain diseases or even mental disorders). Consistently, we have verified here that brain phenomenology is not properly described within BG statistics (i.e., *q* = 1). This is by no means surprising since BG statistics generically disregards inter-component long-range correlations and their collective behavior, which is well known in neural systems^1^. In contrast, *q*-statistics has been empirically shown to be a useful generalization of BGSM^12,22–26^. In addition to other quite informative complexity measures and related methodologies applied to neurosciences^27–31^, *q*-statistics hopefully also is useful in the present case. Here, it was applied through a quite simple methodology, using a functional model involving stretched *q*-exponentials which satisfactorily fit the empirical distributions of scalar inter-event intervals (see Fig. 3). Many of these complex systems present $c_q \ne 0$, from basic chemical reactions through quantum tunneling^32^ to financial market behavior^33^, COVID-19 spreading^34^, commercial air traffic networks^35^ We are led to believe that we are dealing with universality classes of complexity, thus revealing, in what concerns information processing and energy dynamics, far more integrative networks than one might a priori expect from neural structures^36^.

By generalizing the BG theory, *q*-statistics shows that it could be a suitable and promising path to explore brain complexity. Our expectancy is that the *q* parameter can be sensitive to different brain/mental states, to brain/mind development, and to neural diversity, perhaps clarifying the boundaries between the normal and the ill brain, including extreme cases such as Alzheimer, Pick, and Parkinson diseases. Consistently, a key outcome of emergence of self-organized new states in complex systems is an adaptive behavior facing environmental constraints^1^. Indeed, the concept of disease has also been related to reduced adaptive capabilities, and to the alteration of complexity^4,37,38^. Along the lines of the seminal philosophical work of G. Canguilhem^3^, normality should be related to the ability to create new rules (i.e., adaptation) instead of living by the same old norms. We intend to further explore, in the future, the neural diversity through the most remarkable paradigm of complexity.

## Data Availability

The raw EEG wavesfrom all subjects are provided at data.mendeley.com under 10.17632/dm3922zmpj.1.
